# Stiffening of the human proximal pulmonary artery with increasing age

**DOI:** 10.14814/phy2.16090

**Published:** 2024-06-17

**Authors:** Edward P. Manning, Priti Mishall, Abhay B. Ramachandra, Abdulrahman H. M. Hassab, Jerome Lamy, Dana C. Peters, Terrence E. Murphy, Paul Heerdt, Inderjit Singh, Sherry Downie, Gaurav Choudhary, George Tellides, Jay D. Humphrey

**Affiliations:** ^1^ Section of Pulmonary, Critical Care, and Pulmonary Medicine Yale School of Medicine New Haven Connecticut USA; ^2^ VA Connecticut Healthcare System West Haven Connecticut USA; ^3^ Department of Anatomy and Structural Biology Albert Einstein College of Medicine Bronx New York USA; ^4^ Department of Ophthalmology and Visual Sciences Albert Einstein College of Medicine Bronx New York USA; ^5^ Department of Mechanical Engineering Iowa State University Ames Iowa USA; ^6^ Department of Surgery (Cardiac) Yale School of Medicine New Haven Connecticut USA; ^7^ Université Paris Cité, INSERM U970, PARCC, APHP Hôpital Européen Georges Pompidou Paris France; ^8^ Department of Radiology Yale School of Medicine New Haven Connecticut USA; ^9^ Department of Public Health Sciences The Pennsylvania State University College of Medicine Hershey Pennsylvania USA; ^10^ Department of Anesthesiology Yale School of Medicine New Haven Connecticut USA; ^11^ Lifespan Cardiovascular Institute, Providence VA Medical Center Providence Rhode Island USA; ^12^ Warren Alpert Medical School, Brown University Providence Rhode Island USA; ^13^ Department of Biomedical Engineering Yale University New Haven Connecticut USA

**Keywords:** age, aging, human, proximal pulmonary artery, pulmonary artery, stiffening

## Abstract

Adverse effects of large artery stiffening are well established in the systemic circulation; stiffening of the proximal pulmonary artery (PPA) and its sequelae are poorly understood. We combined in vivo (*n* = 6) with ex vivo data from cadavers (*n* = 8) and organ donors (*n* = 13), ages 18 to 89, to assess whether aging of the PPA associates with changes in distensibility, biaxial wall strain, wall thickness, vessel diameter, and wall composition. Aging exhibited significant negative associations with distensibility and cyclic biaxial strain of the PPA (*p* ≤ 0.05), with decreasing circumferential and axial strains of 20% and 7%, respectively, for every 10 years after 50. Distensibility associated directly with diffusion capacity of the lung (*R* ^2^ = 0.71, *p* = 0.03). Axial strain associated with right ventricular ejection fraction (*R* ^2^ = 0.76, *p* = 0.02). Aging positively associated with length of the PPA (*p* = 0.004) and increased luminal caliber (*p* = 0.05) but showed no significant association with mean wall thickness (1.19 mm, *p* = 0.61) and no significant differences in the proportions of mural elastin and collagen (*p* = 0.19) between younger (<50 years) and older (>50) ex vivo samples. We conclude that age‐related stiffening of the PPA differs from that of the aorta; microstructural remodeling, rather than changes in overall geometry, may explain age‐related stiffening.

## Graphical abstract

Aging associates with a significant decrease in both distensibility and cyclic biaxial strain of the human proximal pulmonary artery, likely resulting from microstructural remodeling.

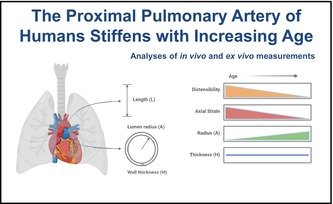

## 1 INTRODUCTION

The proximal pulmonary artery (PPA) is responsible for regulating blood flow from the right ventricle of the heart toward capillaries within the lung where gas exchange occurs. Adverse remodeling of these arteries is associated with various pulmonary pathologies, including chronic obstructive pulmonary disease and pulmonary arterial hypertension (Sanz et al., 2008; Stevens et al., 2012; Vivodtzev et al., 2013; Weir‐McCall et al., 2018; Zhong et al., 2023) as well as the increases in pulmonary arterial pressures that manifest with aging (Horvat et al., 2021; Kane et al., 2016; Lam et al., 2009). These findings were based on in vivo measurements related to pulmonary arterial stiffness. It remains unclear what adverse structural remodeling of the PPA occurs, if any, that may lead to increasing stiffness. There is limited literature on age‐related structural changes of the PPA in humans, the majority of which are ex vivo studies focusing on arterial wall composition and arterial diameter that did not compare these age‐related changes in structure to clinically relevant changes in lung function (Hosoda et al., 1984; Maciejewski et al., 1989; Plank et al., 1980; Warnock & Kunzmann, 1977). Lung function declines with age though the mechanism remains unclear (Vaz Fragoso & Gill, 2012; Vaz Fragoso & Lee, 2012). A large proportion of people over the age of 65 complain of shortness of breath without clear etiology totaling millions of Americans (Miner et al., 2016). Worsening lung function due to “healthy aging” associates with decreased mobility, increased frailty, and increased mortality. We believe that the PPA plays a critical role in age‐related decline in lung function (Fragoso et al., 2012).

The PPA is a large, elastic artery. Elastic arteries connect the heart to end‐organs and are critical for their perfusion and proper function. They augment forward flow of blood while reducing excessive pulsatility in distal circulatory beds; they accomplish this so‐called Windkessel effect primarily by storing elastic energy during systolic distension and subsequently using this energy during diastole to recoil the vessel and augment blood flow. Large arteries achieve this function via their wall elasticity (Ferruzzi et al., 2018), that is, the ability of the wall to return to its original shape with minimal energy dissipation when the load is reduced or removed. This functionality arises primarily through numerous elastic laminae in the media and to a lesser extent through the collagen fibers in the media and adventitia (Townsley, 2012). In addition to arterial wall elasticity, large arteries, such as the PPA, have large luminal diameters to reduce the resistance to the flow of blood from the heart. As a result, the ability of large vessels to functional effectively is often associated with their relationship of wall thickness to luminal diameter (Komutrattananont et al., 2019).

Stiffening has come to be regarded clinically as a loss of pressure‐induced distensibility that consequently impairs the ability of elastic arteries to efficiently store and use energy during the cardiac cycle. This loss of elasticity exerts a negative effect on both circulation and end‐organ function. In the systemic circulation, aortic stiffening is associated with hypertension and aging, which can contribute to end‐organ dysfunction including chronic kidney disease, left ventricular failure, and stroke (Chirinos et al., 2019). This stiffening of arteries also increases pulsatility of blood flow into the arterioles, which results in excessive energy delivery into the capillaries of end‐organs as well as abnormal ventricular‐arterial coupling. Both of these effects may ultimately lead to heart failure among other sequelae (Boutouyrie et al., 2021; Cooper et al., 2016). Similar phenomena have been shown in the pulmonary circulation in a population of patients with pulmonary artery hypertension and right heart failure with preserved ejection fraction (Oakland et al., 2021).

The structural stiffness of an artery depends on two contributors: overall geometry, particularly luminal radius and wall thickness, and intrinsic material stiffness, which results from the composition and microstructural organization of wall constituents. As an example, a thick‐walled materially compliant vessel can have the same structural stiffness as a thin‐walled materially stiff vessel. Whereas material stiffness can affect mechanobiological responses of intramural cells (Humphrey & Schwartz, 2021), it is the structural stiffness that relates directly to distensibility and dictates the hemodynamics (Obeid et al., 2017). To be consistent with clinical usage, we hereafter use the term stiffness to imply structural stiffness.

Stiffening of large arteries can result from adverse wall remodeling, a process that occurs in part in response to changes in mechanical loads with increasing age (Rachev, 2003; van Asten et al., 2023). Local biomechanical changes in the walls of large arteries alter hemodynamics, which consequently change the mechanical loads to which arterial walls are exposed over time (Humphrey et al., 2016). This can create a positive feedback loop that can negatively affect both circulation and end‐organ function. We know that lung function declines as we grow older (Fragoso et al., 2012; Vaz Fragoso & Lee, 2012) and that a large proportion of shortness of breath in the older population remains unexplained (Miner et al., 2016). It is reasonable to hypothesize that both microstructural changes (i.e., the composition and organization of structural proteins and glycoproteins) and morphological changes (i.e., wall thickness and luminal diameter) in the PPA associate with age‐related changes in its overall function (resilience). For this reason, it is important to elucidate what role the adverse remodeling of the PPA plays in the age‐related decline of lung function. We hypothesize that the human PPA stiffens as we age, resulting in localized biomechanical changes that ultimately compromise lung function. To test this hypothesis, we analyzed how strain from in vivo imaging of the PPA using cardiac MRI and how structural properties of the walls of the PPA from ex vivo samples change with increasing age. We further explored associations between aging and remodeling of the PPA as measured by changes in distensibility, biaxial wall strain, wall thickness, vessel diameter, and arterial wall composition. By associating age‐related changes in structure with age‐related decline in cardiopulmonary function, we believe that this study is provides new insights into potential mechanisms for the decline in lung function that accompanies “healthy aging.”

## 2 METHODS

To analyze age‐related changes in the structure and function of the PPA, we combined in vivo studies of patients and ex vivo studies using tissue from cadavers and organ donors. The in vivo analyses of arteries included the in vivo loading condition, whereas the ex vivo analyses of arterial tissue were performed under unloaded conditions. Given the limited data in the literature, we performed this exploratory study as an initial test of our hypothesis that stiffening of the human PPA associates with increasing age. IRB approval was obtained from Yale University for data collection and tissue measurements (2000024783, 200002261, 2000020632).

### 2.1 In vivo imaging

**FIGURE 1:**
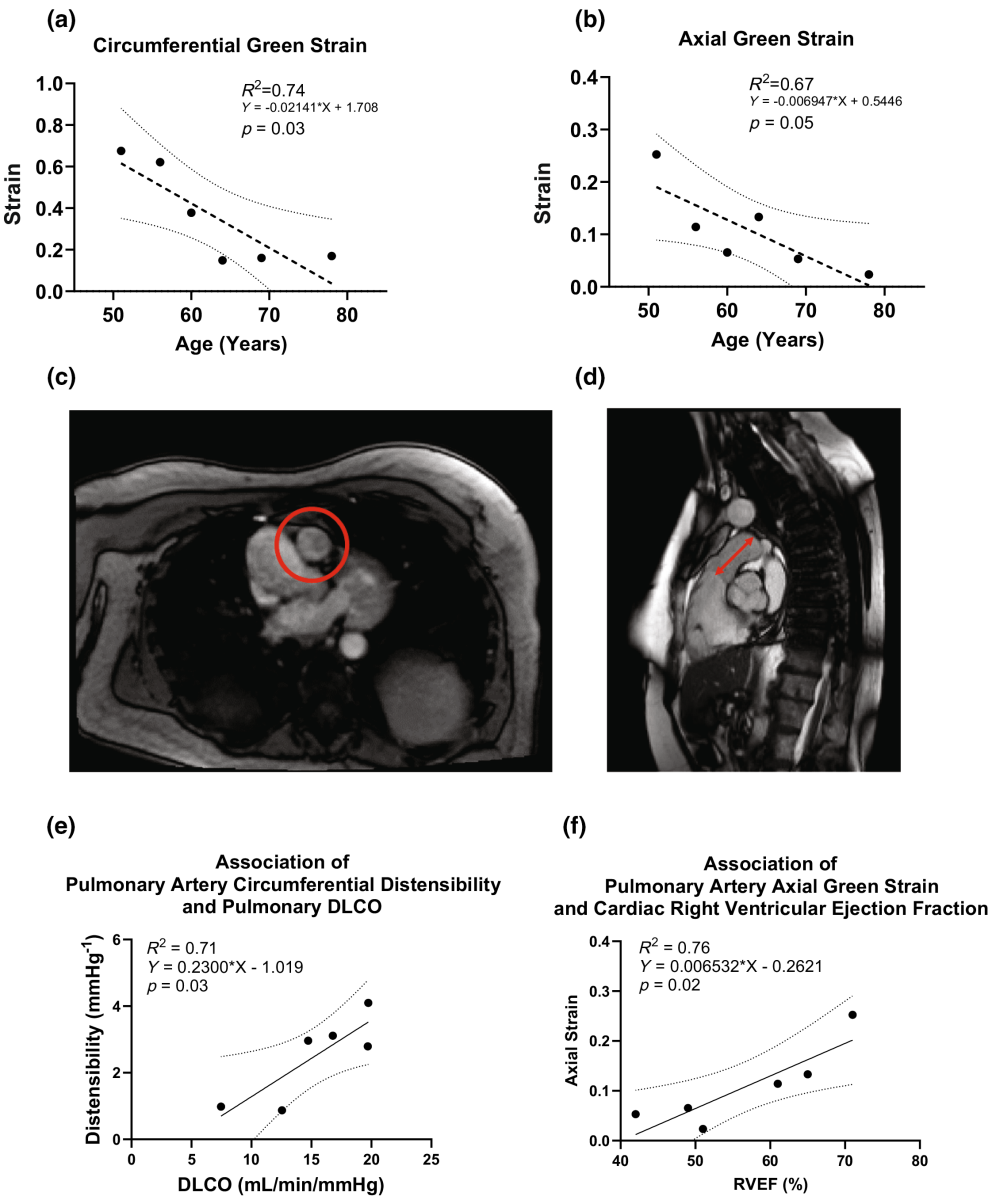
In vivo analyses: Aging exhibits significant negative associations with distensibility and cyclic biaxial strain, characteristics that associate with impaired lung and cardiac function, respectively. Cardiac MRI in vivo measures of circumferential (panel a) and axial (panel b) strain suggest functional decline of the human main pulmonary artery in the circumferential (panel c) and axial (panel d) directions as a function of age, respectively. Distensibility associates with age‐related impairment of lung function (as measured by diffusion capacity during pulmonary function testing, panel e) and axial strain associates with right ventricular dysfunction (as measured by right ventricular ejection fraction calculated from cardiac MRI, panel f). The same patients are represented in all measurements. Pulmonary arterial pressure measurements used to calculate distensibility were obtained from right heart catheterization that was performed on the same day as the cardiac MRI. Pulmonary function tests were performed on separate days within several weeks of the date on which cardiac MRI was performed. Data for these individuals (*n* = 6) are shown in the Table 1. We used simple linear regression to model circumferential strain and axial strain on age in years (panels a and b). We used simple linear regression to separately model DLCO and RVEF on distensibility and axial strain, respectively (panels e and f).

To analyze the mechanical strain experienced by the pulmonary arteries, we used cardiac MRI studies from patients (*n* = 6) who were seen at Yale‐New Haven Hospital (YNHH) in the Pulmonary Vascular Disease clinic. Written informed consent was obtained from these patients to use their data. Characteristics of these patients are included in the Table 1. Patients underwent right heart catheterization and cardiac MRI on the same day. MRI was performed on a Siemens 3 T Scanner using cine acquisitions, showing the cross‐sectional and the oblique sagittal views (Figure 1) of the PPA. The cine acquisitions had the following average scan parameters: 8–10 s breath‐hold, bSSFP, acquisition matrix = 157 × 157, repetition time = 2.6 ms, echo time = 1.29 ms, flip angle = 35° voxel size = 1.88*1.88*6–8 mm, 30 frames 36 ms temporal resolution. Data were analyzed from four patients with mean pulmonary arterial pressure (mPAP) less than 20 mmHg and from two patients with mPAP equal to 25 mmHg with pulmonary vascular resistance less than 3.0 Woods units and pulmonary artery wedge pressure more than 13 mmHg (mild Group II pulmonary hypertension secondary to volume overload from heart failure with preserved ejection fraction) (Simonneau et al., 2019). No patients were treated with vasodilators. Pulmonary artery strain was analyzed in two dimensions—the circumferential direction that encircles the artery in a cross‐sectional plane (relates to distensibility) and the axial direction that runs parallel to the length of the artery (reveals extensibility).

**TABLE 1:** Demographic information for in vivo participants (patients who underwent right heart catheterization and cardiac MRI) and ex vivo samples (cadavers and donors). Data are listed by age within each subsection. Corresponding data (Distensibility as well as circumferential and axial strain of the proximal pulmonary artery, diffusion capacity (DLCO), right ventricular ejection fraction, arterial measurements, etc.) are also presented for reference. Empty cells indicate that data were not available for those ex vivo samples.

In vivo participants (*n* = 6), mean age = 63, range = 51–78									
Age (years)	Sex	Ethnicity	History	Smoke (pack years)	Distensibility (mmHg^−**1**^)	Circ. Green strain	Axial Green Strain	DLCO (mL/min/mmHg)	RVEF (%)
51	F	White, not Hispanic	Raynaud, ILD, hypothyroid	0	4.10	0.68	0.25	19.74	71
56	F	White, not Hispanic	HTN, GERD, HLD	3	3.11	0.62	0.11	16.79	61
60	M	White, not Hispanic	CREST, celiac, GERD, ILD, CM	6	2.96	0.38	0.07	14.73	49
64	F	White, not Hispanic	None	0	2.79	0.15	0.13	19.70	65
69	F	White, not Hispanic	Breast cancer, HLD, OSA	0	0.98	0.16	0.05	7.47	42
78	F	White, not Hispanic	HTN, HLD, CM, OSA	0	0.87	0.17	0.02	12.55	51

Ex vivo samples (*n* = 21)							
Age (years)	Sex	History	Length (mm)	Inner radius (mm)	Wall thick‐ness (mm)	H/A	Cause of death
Cadavers (*n* = 8), mean age = 80, range = 65–89							
65	M	Venous thrombosis, HTN, CAD	39.0	13.8	1.03	0.074	Stroke
72	M		57.7				Cardiogenic shock
76	M	HTN, cerebral palsy	45.7	15.4	0.94	0.061	Sepsis, pneumonia
78	F	Advanced dementia	49.0	14.0	1.15	0.082	Advanced Dementia
83	F	Metabolic encephalopathy, CAD	67.3	12.3			Respiratory Failure
86	M	Parkinsons	43.7	11.4	0.82	0.072	Parkinsons
88	M	Cardiac failure	66.3		1.01		CAD, CM
89	F		58.7	15.5			Sepsis, UTI

Age (years)	Sex	Ethnicity	History	Inner radius (mm)	Wall thick‐ness (mm)	H/A	Histology	Cause of death
Donors (*n* = 13), mean age = 54, range = 18–77								
18	M	White, not Hispanic	None				Yes	Suicide
32	M	White, not Hispanic					Yes	Cardiogenic shock
33	M	African American, not Hispanic	CAD, Obesity				Yes	
42	F	White, not Hispanic	HTN	11.26	1.04	0.092	Yes	Stroke
42	M	White, not Hispanic			1.20		No	Bowel perforation
51	F	Hispanic or Latino	Obesity	10.09	1.14	0.113	Yes	Anoxia, seizure
62	F	African American, not Hispanic	None	13.05	0.93	0.071	Yes	Anoxia, Overdose
63	M	White, not Hispanic	HLD	9.64	1.07	0.110	Yes	Stroke
67	M	White, not Hispanic		13.78	0.70	0.051	Yes	
68	M	White, not Hispanic			1.32	0.082	No	Advanced Dementia
70	M	White, not Hispanic			1.06	0.092	No	Sepsis, UTI
72	F	White, not Hispanic	None	12.3	0.87	0.071	Yes	
77	F	White, not Hispanic	CAD, Obesity	13.93	1.5	0.108	Yes	Stroke

Abbreviations: CAD, coronary artery disease; CM, cardiomyopathy; CREST, calcinosis, Raynaud's phenomenon, esophageal dysfunction, sclerodactyly, telangectasia; GERD, gastroesophageal reflux; HLD, hyperlipidemia; HTN, hypertension; ILD, interstitial lung disease; OSA, obstructive sleep apnea.

Maximum (systolic) and minimum (diastolic) luminal diameter (d) of the main pulmonary artery were measured from which distensibility (D) was calculated as follows:

$$D=\frac{d_{\max}-d_{\min}}{d_{\min}\left(P_{\mathrm{sys}}-P_{\mathrm{dia}}\right)},$$

where *P*_sys_ is the systolic pulmonary artery pressure measured by right heart catheterization and *P*_dia_ is the diastolic pressure. Importantly, local measures of pulse wave velocity (PWV) can also be estimated via the Bramwell‐Hill relation as:

$$\mathrm{PWV}=\sqrt{\frac{1}{\rho D},}$$

where ρ is the mass density of the blood.

To quantify the ability of the pulmonary artery to “distend” or “extend” under physiological loading, we also computed circumferential and axial values of strain:

$$\text{Circumferential Green Strain}=0.5\left(\;\frac{d_{\max}^{2}}{d_{\min}^{2}}-1\right),$$

$$\text{Axial Green Strain}=0.5\left(\;\frac{l_{\max}^{2}}{l_{\min}^{2}}-1\right),$$

where *max* and *min* again refer to systolic and diastolic, d is luminal diameter and l is the distance from the root of the pulmonary artery to the bifurcation of the main pulmonary artery. Note that Green strains provide accurate values regardless of the magnitude of the strain, which is preferred relative to the linearized strain which is given as $d_{\max}/d_{\min}-1$ and $l_{\max}/l_{\min}-1$ in circumferential and axial directions, respectively. Strain was then analyzed as a function of age.

One expects that changes in the circumferential direction associate with changes in hemodynamics, such as pulse wave velocity, which in turn could alter the capillary‐alveolus structure (Tan, Madhavan, et al., 2014; Tan, Tseng, et al., 2014). Alterations in lung structure at the level of the capillary‐alveolus interface would interfere with diffusion capacity of the lung—the lung's ability to transfer gas between the air and blood in the pulmonary circulatory system, which we observed through measures of diffusing capacity of the lungs for carbon monoxide (DLCO). For this reason, we analyzed changes in distensibility with respect to changes in DLCO. We also explored the relationship between changes in axial strain of the pulmonary artery and right ventricular function (Chirinos et al., 2019; Tan, Madhavan, et al., 2014; Tello et al., 2019; Zhong et al., 2023).

### 2.2 Statistical analysis of in vivo data

We first listed the demographics of the six in vivo participants along with their measured values of distensibility, circumferential strain, axial strain, DLCO, and right ventricular ejection fraction (RVEF). We then used simple linear regression to model circumferential strain and axial strain on age in years. To demonstrate how these age‐related changes in strain affect lung function, we also conducted simple linear regression to separately model DLCO and RVEF on distensibility and axial strain, respectively. The regression analyses were made using GraphPad PRISM Version 10.1.2 where significance was defined as any *p*‐value <0.05.

### 2.3 Ex vivo measurements

To analyze structural properties of the walls of the main pulmonary artery, tissue samples were obtained from de‐identified donors from the Anatomy Dissection Laboratory of the Albert Einstein College of Medicine, organ donors whose hearts were not used for clinical transplantation from the New England Donor Services, and National Disease Research Interchange (USA). IRB approval for tissue research was obtained from these institutions. Morphometric analyses of human pulmonary arteries were performed by measuring both wall thickness and luminal diameters of the main pulmonary artery using Vernier calipers, recognizing that these measurements were made under unloaded conditions. All measurements were made in triplicate, and mean values were regressed on increasing age. The ratio of wall thickness (H) to inner radius (A) of the arteries was also regressed on increasing age.

To analyze the composition of the arterial wall, samples were cut in cross‐section, embedded in paraffin, and stained with Hematoxylin and Eosin (H&E), Elastin van Gieson (EVG), and Masson's Trichome (MTC) by Yale Research Histology. The proportion of collagen and elastin within each sample was calculated using color threshold analyses in Matlab as described previously (Bersi et al., 2017; Manning et al., 2021). Briefly, images were analyzed by pixel‐level thresholding to calculate area fractions of elastin from VVG and collagen from MTC, a reproducible means of quantifying composition of walls of large arteries (Bersi et al., 2017; Ferruzzi et al., 2015; Manning et al., 2021). Samples were binned into two groups to analyze the change of arterial wall composition as a function of age. The young group comprises samples from individuals <50 years; the older group comprises samples from individuals >50 years.

### 2.4 Statistical analysis of ex vivo data

All available sociodemographic data corresponding to the eight cadavers and the 13 organ donors were tabulated. We then used simple linear regression of the following characteristics of the PPA on age using as many of the ex vivo samples that allowed evaluation: length (eight cadavers), inner radius (six cadavers and seven donors), wall thickness (five cadavers and 10 donors), and the ratio of wall thickness to inner radius (four cadavers and nine donors). The resulting regression lines and their 95% confidence intervals were plotted. To provide some reference, in each case the six data points measured from the in vivo sample were superimposed on the regression plots. We note that the six in vivo data points were not used in the fitting of the regression line. Rather they are added to contrast the line fitted on the ex vivo data with the in vivo data points. Finally, the proportions of elastin and collagen in the same age‐based groups were compared using a chi‐square test. The same statistical software and definition of significance were retained from the in vivo data analysis.

## 3 RESULTS

### 3.1 In vivo imaging

There were six in vivo participants. Their mean age was 63 (range = 51–78), and the majority shared similar cardiopulmonary co‐morbidity profiles (Table 1). The clinical metric of distensibility *D* decreased significantly with age (Figure S1a, *p* = 0.003). Likewise, the calculated pulse wave velocity increased significantly with age (Figure S1b, *p* = 0.006). Values of biaxial Green strain were computed for all six patients and regressed on increasing age. Both the circumferential strain (Figure 1a), based on cross‐sectional measurements from cardiac MRI (Figure 1c), and the axial strain (Figure 1b), based on long‐axis measurements from cardiac MRI (Figure 1d), decreased significantly with increasing age (*p* ≤ 0.05). The coefficients for circumferential and axial strain per year were approximately 0.02 and 0.007, respectively (refer to equations in Figure 1a,b, respectively). These small effect sizes correspond to yearly increments in age and to provide some perspective of their longitudinal impact: every 10 additional years of age represent decreases of approximately 20% in circumferential strain and 7% in axial strain. There was also a trend toward lower diffusion capacity with increasing age (Figure S1c, *p* = 0.17). Figure 1e shows a statistically significant association between increasing DLCO and increasing distensibility (*R* ^2^ = 0.71, *p* = 0.03). Figure 1f similarly shows a significant association between increasing right ventricular ejection fraction and increasing axial strain (*R* ^2^ = 0.76, *p* = 0.02).

### 3.2 Ex vivo measurements

**FIGURE 2:**
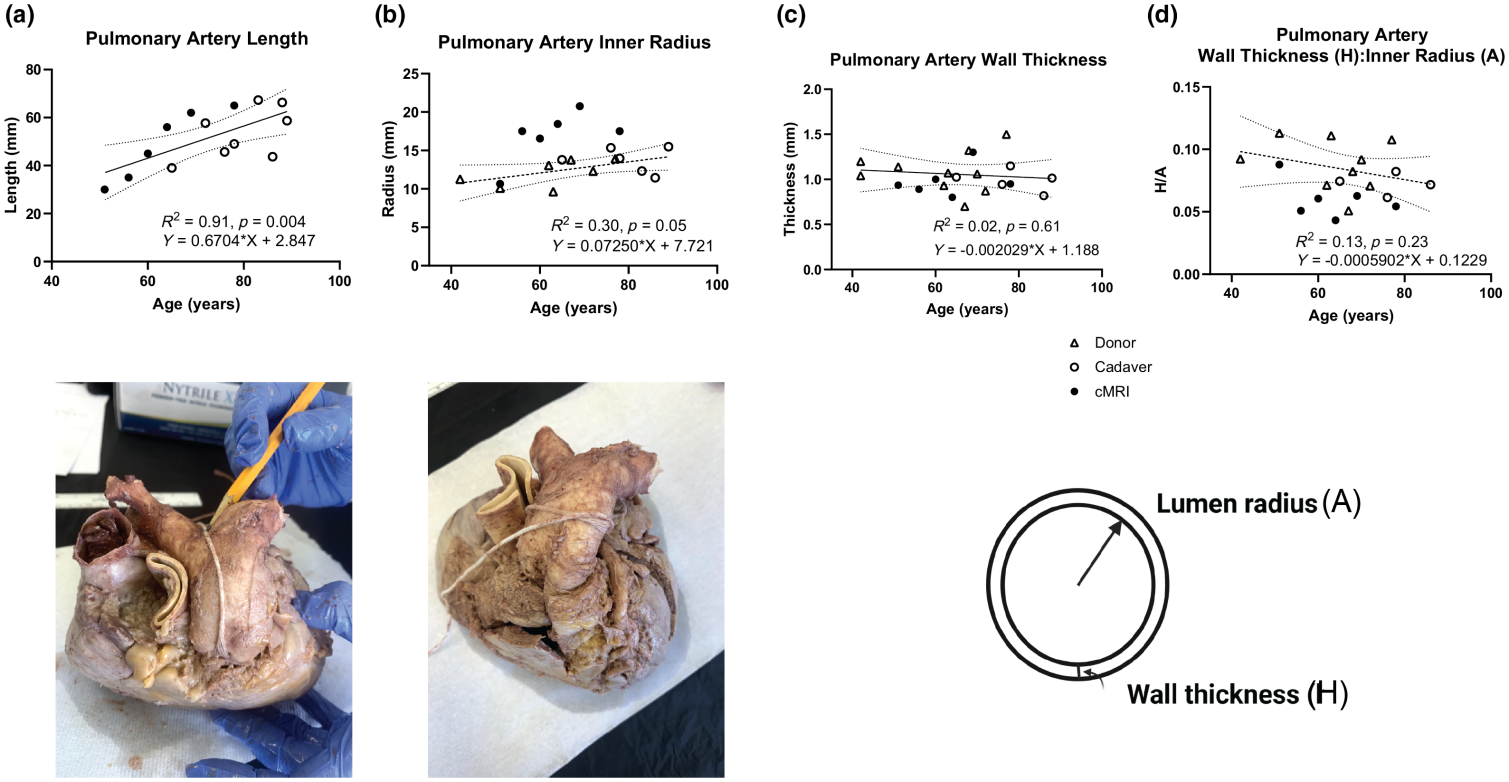
Ex vivo analyses: Changes in the overall structure of the proximal pulmonary artery as a function of age. The length of the main pulmonary artery increases significantly with age when measured from the root of the artery at which the artery exits from the right ventricle of the heart to the bifurcation of the main pulmonary artery into to the right and left pulmonary arteries (panel a, representative length measurement indicated by white string). The radius of the main pulmonary artery increases with age (panel b, representative outer circumferential measurement indicated by white string), whereas, the thickness of the human main pulmonary artery does not change significantly with age (panel c). Thus, there is a gradual decrease in wall thickness‐to‐lumen radius ratio (H/A, panel d). This suggests that the microstructural remodeling of the arterial wall of the human pulmonary artery is a major contributor to the overall stiffness of the artery whereas wall thickness is not. Measurements taken from cardiac MRI were in vivo, therefore, under physiologic load. Measurements from donor and cadaver tissue were measured ex vivo in unloaded conditions. Best‐fit lines reflect ex vivo measurements; in vivo measurements are included for reference. Supplfigure s lines are the 95% confidence intervals. We used simple linear regression of the following characteristics of the PPA on age using as many of the ex vivo samples that allowed evaluation: length (eight cadavers), inner radius (six cadavers and seven donors), wall thickness (five cadavers and 10 donors), and the ratio of wall thickness to inner radius (four cadavers and nine donors). The resulting regression lines and their 95% confidence intervals were plotted. To provide some reference, in each case the six data points measured from the in vivo sample were super‐imposed on the regression plots. We note that the six in vivo data points were not used in the fitting of the regression line. Rather they are added to contrast the line fitted on the ex vivo data with the in vivo data points. Symbols: hollow triangles indicate ex vivo data from donor samples; hollow circles indicate ex vivo data from cadavers; solid black circles indicate in vivo data from cardiac MRI.

**FIGURE 3:**
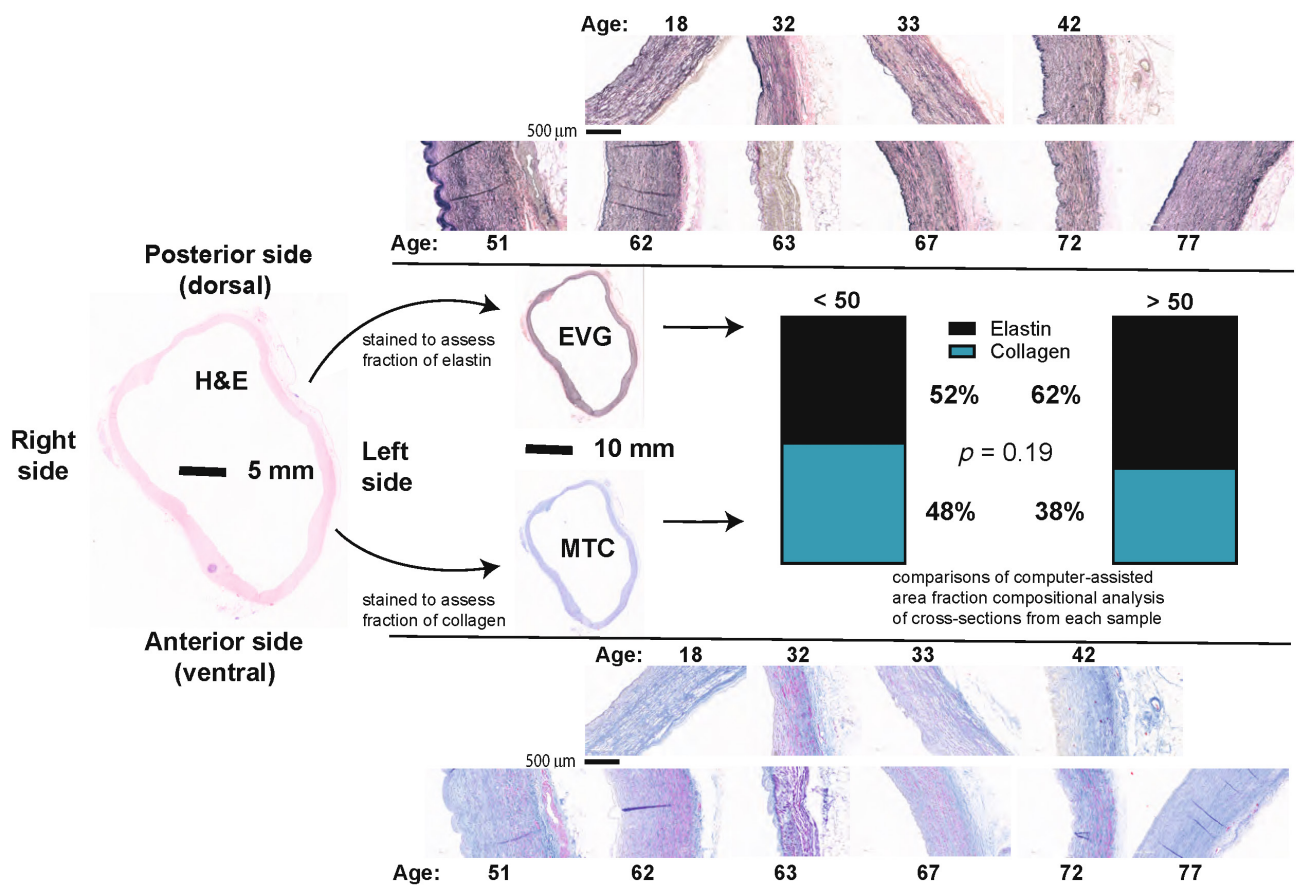
Proportions of elastin and collagen in older (>50) versus young (<50) samples. There is no significant change in the proportion of elastin to collagen (*p* = 0.19) when comparing older (*n* = 6) with young (*n* = 4) donor samples. The proportions of elastin and collagen in the same age‐based groups were compared using a chi‐square test.

To investigate morphological changes in the wall of the PPA that might explain the changes observed in circumferential and axial strain from in vivo imaging, in our ex vivo samples we analyzed age‐related changes of length, wall thickness, luminal radius, and the relationship between thickness and radius. There were 21 samples from cadavers (mean age 80, range = 65–89) and 13 from donors (mean age 54, range = 18–77). Clinical information was limited on these samples but is shown in the Table 1. We observed a significant increase of the length of the artery with aging (Figure 2a, *p* = 0.004). The age‐related remodeling of the PPA resulting in increases in length also associated with the age‐related decrease in axial strain. There was a significant increase in luminal radius with age (Figure 2b, *p* = 0.05), but no corresponding change in mean wall thickness (Figure 2c, H_μ_ = 1.19 mm, SD = 0.19 mm, *p* = 0.61). The wall‐to‐lumen ratio (H/A), therefore, trends downward with age (Figure 2d). There was no statistically significant difference in the proportions of elastin and collagen (*p* = 0.19) between the young (age < 50 years) and old (>50 years) subgroups of the ex vivo samples that might explain these changes (Figure 3).

## 4 DISCUSSION

In this exploratory study, we analyzed the distensibility and cyclic biaxial strain of proximal pulmonary arteries using cardiac MRI from in vivo samples and morphological and microstructural characteristics of arteries from ex vivo donors between the ages of 18 and 89 years. We observed age‐related decreases in distensibility and cyclic biaxial strain that suggest an age‐related attenuation in the function of the PPA. These functional changes also reflect a steady process of age‐related structural stiffening. This age‐related rise in stiffness appears to result primarily from an increase in adverse microstructural remodeling (most likely due to organization of mural constituents) rather than changes in the geometry (lumen radius and wall thickness).

Stiffening of the proximal, extralobar pulmonary arteries can have large effects on blood flow in the pulmonary circulation (Milnor et al., 1969; Tan, Madhavan, et al., 2014). Such stiffening negatively impacts the primary biomechanical function of those proximal arteries—to store elastic energy during systolic distension and subsequently to use this energy to work on the blood to augment forward flow in a smooth, controlled manner. One consequence of this stiffening is quantified as pulse wave velocity, with increases therein reflecting faster propagation of pulse waves and earlier reflections from distal sites—traits of poor circulatory hemodynamics. Importantly, impaired pulmonary hemodynamics can impair lung function and RV function (Milnor et al., 1969; Nichols et al., 2008; Oakland et al., 2021; van den Berg et al., 2018). The measured age‐related worsening of PPA distensibility suggests that both RV afterload and pulsatility within the pulmonary microcirculation increase with age (Horvat et al., 2021); both of these hemodynamic changes are known to impair lung function (Tan, Tseng, et al., 2014). Pulmonary arterial stiffening associates with several types of lung disease, including chronic obstructive pulmonary disease and pulmonary hypertension (Vivodtzev et al., 2013; Weir‐McCall et al., 2018). However, this is the first ex vivo study to correlate structural changes in the PPA with age‐related decline in lung function.

There is a well‐described age‐related decline in lung function featuring significant decreases in diffusion capacity (Park et al., 1986) that commonly presents as clinical dyspnea. Even though it associates with decreased activity, increased frailty, and increased mortality, dyspnea is a common geriatric syndrome that often goes unexplained in older individuals (Fragoso et al., 2012; Miner et al., 2016). Our study suggests a direct association between age‐related stiffening of the PPA and functional decline of the lungs, a mechanism that has attracted little attention in the literature. Our finding that the length of the pulmonary artery increases with aging whereas strain decreases supports the hypothesis that age‐related stiffening of the main pulmonary artery in humans results in large part from microstructural remodeling. Recently, it was suggested that decreased axial strain (extensibility) of the PPA associates with poor prognosis in patients with pulmonary arterial hypertension (Zhong et al., 2023). Our study suggests that decreased axial strain of the PPA also associates with aging and proposes a potential tissue‐level mechanism for decline in right ventricular function, a phenomenon that has been observed in studies such as the MESA study (Chatterjee et al., 2017). In a similar manner, the decreased axial strain that associated with increased afterload on the right ventricle may also impair right ventricular function (Boutouyrie et al., 2021; Chirinos et al., 2019; Cooper et al., 2016; Tan, Madhavan, et al., 2014; Tello et al., 2019; Zhong et al., 2023). In summary, the characteristics of age‐related stiffening of the main pulmonary artery in humans may associate with age‐related dysfunction of the lungs and the right ventricle.

As elastin and collagen are the two major structural constituents of the extracellular matrix in the PPA, it seems reasonable to assume that their overall quantity or ratio may explain the observed functional changes (Burgstaller et al., 2017). However, we found no age‐related change in the amount of fibrillar collagen within the PPA. This differs from findings in the aorta, in which loss of intact elastic fibers with age associates with increases in the amount of collagen in the outer (adventitial) layer (Geest et al., 2005; Hosoda et al., 1984), with both contributing to the greater stiffness of older aortas (Chirinos et al., 2019; Mammoto et al., 2022; Roldan et al., 2021). In the human main pulmonary artery, excessive adventitial collagen appears not to play a critical role in the age‐related stiffening. Consistent with previous research, we observed no age‐related change in the proportion of elastin or the ratio between elastin and collagen in the PPA (Hosoda et al., 1984; Plank et al., 1980). We therefore suspect that remodeling of the extracellular matrix may explain the age‐related changes in function that we observe. In mice, we have observed stiffening of the PPA due to chronic hypoxia that is characterized by reorientation of the adventitial collagen fibers rather than an altered quantity of such fibers (Manning et al., 2021). This microstructural reorganization may be a potential mechanism of age‐related material stiffening in the human PPA that requires future investigation. Another contributor to stiffening may be an age‐related increase in the cross‐linking of collagen as has been observed in the aorta (Murtada et al., 2021; Spronck et al., 2020). Unfortunately we were not able to assess either the orientation or cross‐linking of collagen fibers in this study.

Direct measurements of stiffness of human proximal pulmonary arteries are scarce but have been performed in large animals and mouse models (Chemla et al., 2018; Manning et al., 2021; Reusser et al., 2012). Clinical inferences of structural stiffening based on measures of distensibility have been associated with pulmonary hypertension in humans (Chemla et al., 2018; Gan et al., 2007) and aging (Horvat et al., 2021). This exploratory study provides foundational data for computational modeling (Jiang et al., 1994) and to guide future studies. We believe that microstructural remodeling of the constituents of the arterial wall plays a significant role in age‐related stiffening of the PPA. Since the wall thickness of the PPA does not appear to increase with normal aging, we believe that future clinical or research studies seeking to measure pulmonary arterial stiffness will likely not benefit from focusing on differences in wall thickness or perivascular support. Similarly, investigating the overall composition of the proximal artery, emphasizing quantities of elastin, collagen, or the ratio between them, will provide little benefit. This is consistent with earlier research that found that elastin density in the pulmonary trunk changed little with age and concluded that distensibility is likely the result of altered physical properties of the arterial wall and its components rather than the changes in quantity of wall components (Plank et al., 1980). Our study supports that conclusion. Additional components of the extracellular matrix warrant investigation, and additional imaging techniques focusing on the quality rather than quantity of extracellular matrix components are likely necessary. Techniques including second harmonic generation to image large volumes of collagen without the need for staining appear ideal (Cavinato et al., 2020, 2021). Histologic imaging focusing on characteristics of collagen such as fiber undulation and orientation also appear likely to be beneficial (Macoska et al., 2019).

Lastly, increasing wall‐to‐lumen ratio under loaded conditions has been associated with arterial structural stiffening, arterial function, and functional decline of the organs with which they are associated (Ritt & Schmieder, 2009; Salvetti et al., 2014; Schmieder & Ritt, 2012; Thijssen et al., 2011). It was also shown to increase in the aorta with aging (da Silva et al., 1999; Komutrattananont et al., 2019; Sawabe et al., 2011). It appears that we are the first to show that wall‐to‐lumen ratio of the PPA remains unchanged, or possibly decreases, with aging. This further supports our belief that microstructural remodeling plays a critical role in increasing stiffness in age‐related impairment of the PPA.

A major limitation of this study is the sample size used for each experiment. It is logistically difficult to perform right catheterization pressure measurements on the same day as imaging is obtained by cMRI. We benefited from the use of data obtained in this manner from individuals referred to our Pulmonary Vascular Disease clinic. By the nature of their referral to this clinic, very few individuals had non‐pathological vasculature. While we were left with a small sample to evaluate the effects of healthy aging, the changes were large enough to observe. However, a larger study with sufficient sample size to control for subjects' health status, including cardiometabolic risk factors such as systemic hypertension, diabetes, and hyperlipidemia, is warranted. Another limitation is that ex vivo measurements were made in the unloaded state while in vivo measurements were at in situ stretches and pressures. Thus, our in vivo sample size is small, which results in an underpowered study that renders trends of in vivo measurements susceptible to outliers and confounders such as cardiometabolic risk factors including systemic hypertension, diabetes, coronary artery disease, and hyperlipidemia. A larger study comparing ex vivo and in vivo measurements can improve the power of future studies as discussed above and is currently underway. We also aim to gather larger physical samples of tissue in the future to perform biochemical experiments to better characterize the extracellular matrix of the arterial wall, including hydroxyproline assay to quantify collagen content and Western Blot analyses of matrix proteins such as collagen, elastin, fibronectin, and others. Our current study is limited by the lack of biochemical measurements of these proteins.

While this complicates direct comparison of some measures, we yet observed similar trends in the structural and functional metrics that support that proximal pulmonary arteries stiffen as we grow older. Another limitation in this study involves the nature of tissue samples. Cadaveric samples were embalmed, donor samples were fresh and not fixed, and in vivo samples were living. Despite these differences, there was good agreement across samples for certain values such as wall thickness, and similar trends were observed for other measurements such as lumen radius. Finally, the wall‐to‐lumen radius measurements were made unloaded, which prevents its use as a surrogate for wall stress or stiffness; however, it provides some insight into the changing nature of the material constituting the arterial wall. Further, it provides important data for designing future studies of stiffness of the human pulmonary artery.

In conclusion, across a combination of in vivo and ex vivo samples we observed age‐related stiffening and functional decline of the proximal pulmonary artery in humans. Our results suggest that the mechanistic path of age‐related changes in the PPA may differ markedly from that of the aorta, where changes in overall geometry partially explain its age‐related stiffening. Our findings suggest that localized microstructural remodeling of the PPA wall may be responsible for its age‐related stiffening and corresponding functional decline. Future studies should investigate changes in the extracellular matrix beyond the simple quantification of collagen and elastin to better understand tissue level mechanisms. Related cellular and molecular studies should be pursued as well.

## AUTHOR CONTRIBUTIONS

EPM and PM designed study, performed experiments, and wrote manuscript; ABR performed experiments and edited manuscript; AHMH performed experiments; JL and DP analyzed imaging; TEM designed experiments, interpreted data analysis, and reviewed the manuscript; PH supervised experiments and edited manuscripts; IS and GC designed experiments, analyzed data, and edited manuscript; SD designed experiments, performed experiments, and edited manuscript; GC edited and revised manuscript; GT and JDH designed experiments, supervised experiments, and edited manuscript.

## ETHICS STATEMENT

IRB approval was obtained from Yale University for in vivo data collection and tissue measurements (2000024783, 200002261, 2000020632). Ex vivo measurements samples were obtained from de‐identified donors from the Anatomy Dissection Laboratory of the Albert Einstein College of Medicine, organ donors whose hearts were not used for clinical transplantation from the New England Donor Services, and National Disease Research Interchange (USA). IRB approval for tissue research was obtained from these institutions.

## Supporting information


Figure S1.

## Data Availability

Data for figures has been included in the Table where possible. Additional data is available upon request.
