# Supplementary material for: Stiffening of the human proximal pulmonary artery with increasing age
Source: Physiol Rep. 2024 Jun 17;12(12):e16090. doi: 10.14814/phy2.16090 (PMC11181131; doi:10.14814/phy2.16090)
Supplement: Supplementary file 1 — Figure S1. [file PHY2-12-e16090-s001.docx]

Supplemental Material

**Stiffening of the Human Proximal Pulmonary Artery with Increasing Age**

Edward P. Manning^1,2,*,+^, Priti Mishall^3,4,*^, Abhay B. Ramachandra^5^, Abdulrahman H. M. Hassab^6^, Jerome Lamy^7^, Dana Peters^8^, Terrence E. Murphy^9^, Paul Heerdt^10^, Inderjit Singh^1^, Sherry Downie^3^, Gaurav Choudhary^11,12^, George Tellides^2,6^, Jay D. Humphrey^13^

1 - Section of Pulmonary, Critical Care, and Pulmonary Medicine, Yale School of Medicine, New Haven, CT, USA

2 - VA Connecticut Healthcare System, West Haven, CT, USA

3 – Department of Anatomy and Structural Biology, Albert Einstein College of Medicine, Bronx, NY, USA

4 – Department of Ophthalmology and Visual Sciences, Albert Einstein College of Medicine, Bronx, NY, USA

5 – Department of Mechanical Engineering, Iowa State University, Ames, IA, USA

6 – Department of Surgery (Cardiac), Yale School of Medicine, New Haven, CT, USA

7 - Université Paris Cité, INSERM U970, PARCC, APHP Hôpital Européen Georges Pompidou, Paris, France
8 – Department of Radiology, Yale School of Medicine, New Haven, CT, USA

9 – Department of Public Health Sciences, The Pennsylvania State University College of Medicine, Hershey, PA, USA

10 – Department of Anesthesiology, Yale School of Medicine, New Haven, CT, USA

11 – Lifespan Cardiovascular Institute, Providence VA Medical Center, Providence, RI, USA

12 – Warren Alpert Medical School, Brown University, Providence, RI, USA

13 – Department of Biomedical Engineering, Yale University, New Haven, CT, USA

*contributed equally

+Corresponding author: [edward.manning@yale.edu](mailto:edward.manning@yale.edu), 300 Cedar St, TAC S460, New Haven, CT 06520

**
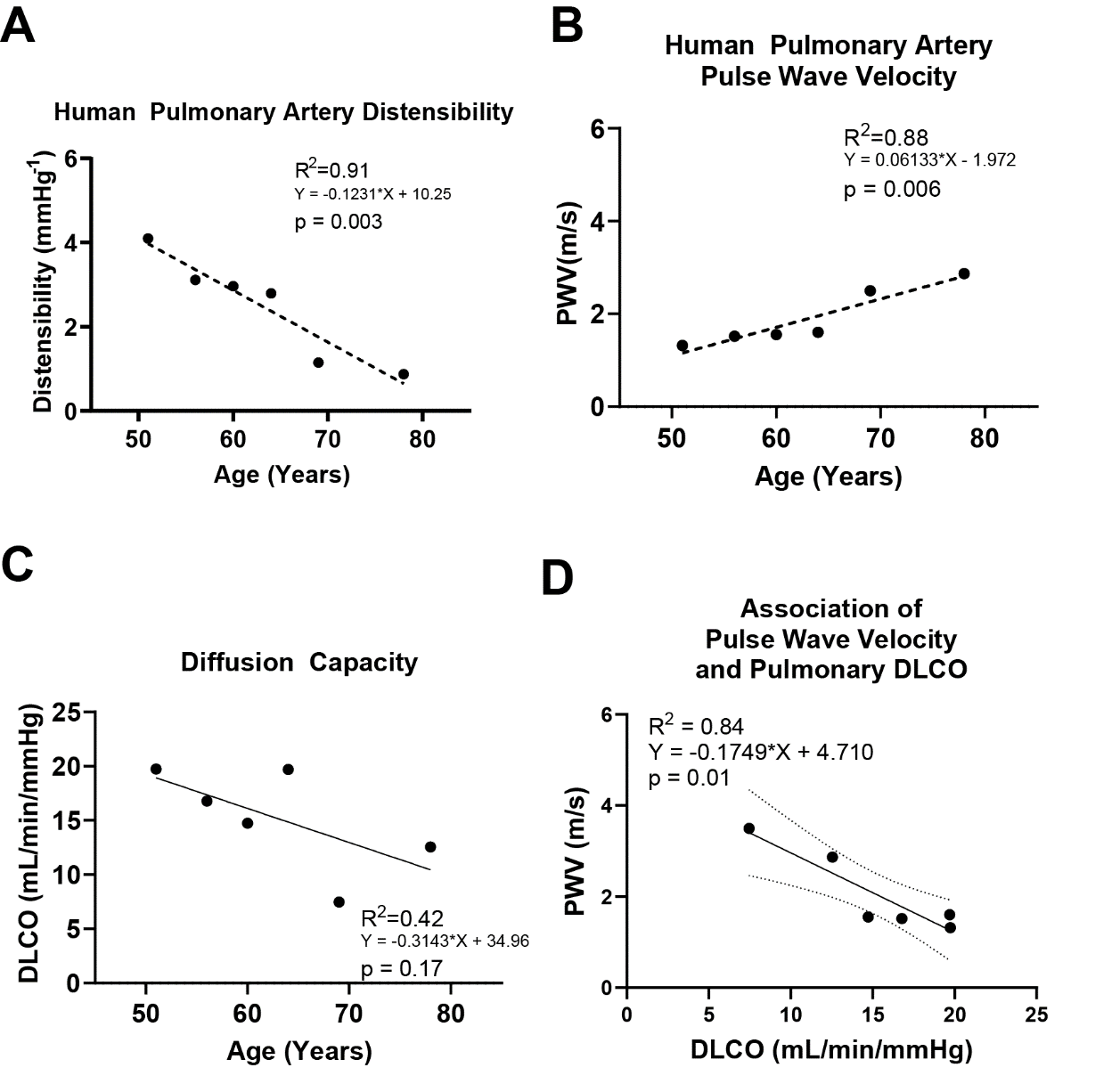
**

**Supplemental Figure 1:** A: Cardiac MRI *in vivo* measure of distensibility suggests a progressive decline of the human main pulmonary artery as a function of age. B: The calculated pulse wave velocity increases significantly with age. C: Pulmonary function test *in vivo* measure of diffusion capacity (DLCO) suggests a decline of gas exchange function in the human lungs as a function of age. D. Association of pulse wave velocity (PWV) and pulmonary DLCO.
